# *Salmonella* serotypes in the genomic era: simplified *Salmonella* serotype interpretation from DNA sequence data

**DOI:** 10.1128/aem.02600-24

**Published:** 2025-02-24

**Authors:** Xiangyu Deng, Shaoting Li, Tongzhou Xu, Zhemin Zhou, Michelle M. Moore, Ruth Timme, Shaohua Zhao, Charlotte Lane, Blake A. Dinsmore, François‑Xavier Weill, Patricia I. Fields

**Affiliations:** 1 Center for Food Safety, University of Georgia 308511 , Griffin, Georgia, USA; 2 School of Biomedical and Pharmaceutical Sciences, Guangdong University of Technology 571332 , Guangzhou, China; 3 Soochow University Cancer Institute, Soochow University 12582 , Suzhou, China; 4 Office of Regulatory Science, Office of Regulatory Affairs, U.S. Food and Drug Administration, Rockville, Maryland, USA; 5 Center for Food Safety and Applied Nutrition, U.S. Food and Drug Administration 116043 , College Park, Maryland, USA; 6 Center for Veterinary Medicine, U.S. Food and Drug Administration 621252 , Laurel, Maryland, USA; 7 Division of Foodborne, Waterborne and Environmental Diseases, Centers for Disease Control and Prevention 1242 , Atlanta, Georgia, USA; 8 Institut Pasteur, Université Paris Cité 555089 , Paris, France; Michigan State University, East Lansing, Michigan, USA

**Keywords:** *Salmonella*, serotype, whole genome sequencing

## Abstract

In the era of genomic characterization of strains for public health microbiology, whole genome sequencing (WGS)-enabled subtyping of *Salmonella* provides superior discrimination of strains compared to traditional methods such as serotyping. Nonetheless, serotypes are still very useful; they maintain historical continuity and facilitate clear communication. Genetic determination of serotypes from WGS data is now routine. Genetic determination of rarer serotypes can be problematic due to a lack of sequences for rare antigen types and alleles, a lack of understanding of the genetic basis for some antigens, or some inconsistencies in the White-Kauffmann-Le Minor (WKL) Scheme for *Salmonella* serotype designation. Here, we present a simplified interpretation of serotypes to address the shortcomings of genetic methods, which will allow the streamlined integration of serotype determination into the WGS workflow. The simplification represents a consensus perspective among major U.S. public health agencies and serves as a WGS-oriented interpretation of the WKL Scheme. We also present SeqSero2S, a bioinformatics tool for WGS-based serotype prediction using the simplified interpretation.

**IMPORTANCE:**

The utility of *Salmonella* serotyping has evolved from a primary subtyping method, where the need for strain discrimination justified its complexity, to a supplemental subtyping scheme and nomenclature convention, where clarity and simplicity in communication have become important for its continued use. Compared to phenotypic methods like serotyping, whole genome sequencing (WGS)-based subtyping methods excel in recognizing natural populations, which avoids grouping together strains from different genetic backgrounds or splitting genetically related strains into different groups. This simplified interpretation of serotypes addresses a shortcoming of the original scheme by combining some serotypes that are known to be genetically related. Our simplified interpretation of the White-Kauffmann-Le Minor (WKL) Scheme facilitates a complete and smooth transition of serotyping’s role, especially from the public health perspective that has been shaped by the routine use of WGS.

## INTRODUCTION

Despite substantial progress in foodborne pathogen surveillance and control, *Salmonella* remains a leading cause of foodborne infections in the United States, showing a stalled decline in recent years (1–3). U.S. surveillance of *Salmonella* has been built upon serotypes, also called serovars, a typing scheme established in 1934 (4). Over 90 years of literature and 50 years of U.S. surveillance data based on serotype (5) have contributed to a well-established, serotype-centric understanding of *Salmonella* epidemiology, biology, and evolution. For example, specific serotypes are associated with specific disease syndromes (6), host associations (7), and geographic and/or source distribution (8, 9). Serotypes are integral to *Salmonella* nomenclature and serve as a universal terminology for communicating *Salmonella* surveillance and research.

*Salmonella* serotypes are designated according to the conventions of the White-Kauffmann-Le Minor (WKL) Scheme (10). The scheme is based on the characterization of *Salmonella* strains based on two surface structures, the lipopolysaccharide O antigen and flagellar H antigen. The scheme recognizes 46 major O antigens or serogroups plus several secondary O antigens that are found in specific serogroups. Serogroup antigens are commonly encoded by the *rfb* region on the chromosome and the secondary O antigens that have been characterized are typically encoded by mobile genetic elements. H antigens are proteins that are part of the bacterial flagella. They have been characterized as either single epitopes or multiple epitopes. Multi-epitope H antigens are called H antigen complexes. One example, 1 Complex antigens, share a common epitope “1” and differ by one or more secondary epitopes (e.g., H:1,2; H:1,5; H:1,6; H:1,7; and H:1,5,7). Since the same antigenic types may appear in different taxonomic lineages, subspecies identification is commonly included in serotype designations. More than 2,600 specific combinations of O and H antigens (and sometimes additional markers), each a different serotype (or serovar), were described in the current ninth edition of the scheme, which was published in 2007 and has been updated two times since then (11, 12).

Methods to determine serotypes have evolved from phenotypic determination using antigen-antibody agglutination assays to genetic determination using the genes responsible for serotype (13, 14). Genetic determinants include the *rfb* gene cluster encoding genes involved in the biosynthesis of O group antigen and the *fliC* and *fljB* genes encoding the flagellar antigens. Serotype determination using whole genome sequencing (WGS) data was introduced in 2015 (15, 16). SeqSero2, a tool for predicting serotypes based on the genes responsible for serotype (17), has been validated and is routinely used by the Centers for Disease Control and Prevention (CDC), the Food and Drug Administration (FDA), and the U.S. Department of Agriculture Food Safety and Inspection Service (USDA FSIS). It has been incorporated into NCBI Pathogen Detection (https://www.ncbi.nlm.nih.gov/pathogens) and EnteroBase (https://enterobase.warwick.ac.uk/), and validated for the FDA Bacteriological Analytical Manual (https://www.fda.gov/food/laboratory-methods-food/foods-program-compendium-analytical-laboratory-methods).

While serotypes continue to be important for disease surveillance and public health investigations, the antigenic detail in the WKL Scheme is less critical than it once was because serotyping is no longer our primary subtyping method. With the launch of PulseNet in 1996, pulsed-field gel electrophoresis (PFGE) replaced serotyping as the primary subtyping method for major foodborne pathogens (18, 19). Since 2013 (20), PFGE was gradually superseded by WGS-based subtyping methods, such as methods based on single nucleotide polymorphisms (SNP) (21) and genome-wide multi-locus sequence typing in outbreak detection and investigation, e.g., core genome multilocus sequence typing (cgMLST) (22).

Several challenges complicate the replication of a scheme based on phenotype into a genetic one. Although the genetic basis for many serotypes is well understood, it is unknown for some antigenic types described in the WKL Scheme. Genetic methods such as SeqSero2 and SISTR, a combination of *in silico* serotyping and 7-gene multilocus sequence typing (MLST) (14), may incorrectly call an antigenic type when a close allele is not present in the database. Furthermore, some serotypes in the scheme are based on phenotypic characteristics beyond O and H antigens, and inconsistencies in how variable epitopes are presented in the scheme make it difficult to assign some serotypes.

While it is currently impossible to replicate the phenotypic serotype scheme with genetic methods, it is also unnecessary because highly discriminatory genetic methods are now available to provide differentiation beyond serotype data. Genetic methods better reveal natural population groups, which is fundamental for surveillance. Dropping or combing certain serotypes, such as those based on unstable antigens, for instance, O and H antigens that may be present or absent without relation to phage conversion (designated by square brackets in the WKL Scheme), makes serotypes better represent natural populations. Also, the primary value of serotype data in the modern world—clear communication and historical continuity—is diminished for many of the 2,600+ serotypes that are quite rare. Their names are not familiar to the scientific community and defining biologic or epidemiologic characteristics have not been described. A consensus has been reached among a consortium of CDC, FDA, USDA, and SeqSero2 developers to design and implement a simplified interpretation of the scheme for better and more consistent sharing and communication of *Salmonella* serotypes. The goal of the simplification is a scheme where serotype designations can be determined and validated using currently available sequence data and genetic methods. The simplification is based on our experience of determining, communicating, and curating serotypes in public health settings in the United States. Some of the proposed simplifications are already practiced by U.S. agencies. The simplified interpretation has little to no impact on commonly encountered serotypes in the United States and will allow the full transition of serotype determination from a phenotypic to a genetic method. We provide an implementation of the simplified interpretation, designated as SeqSero2S (https://www.denglab.info/SeqSero2, https://github.com/denglab/SeqSero2S), including additional functionalities to improve genomic prediction of serotypes in general and mitigate potential drawbacks of the simplified scheme.

## RESULTS

### Currently circulating *Salmonella* serotypes

**Fig 1:**
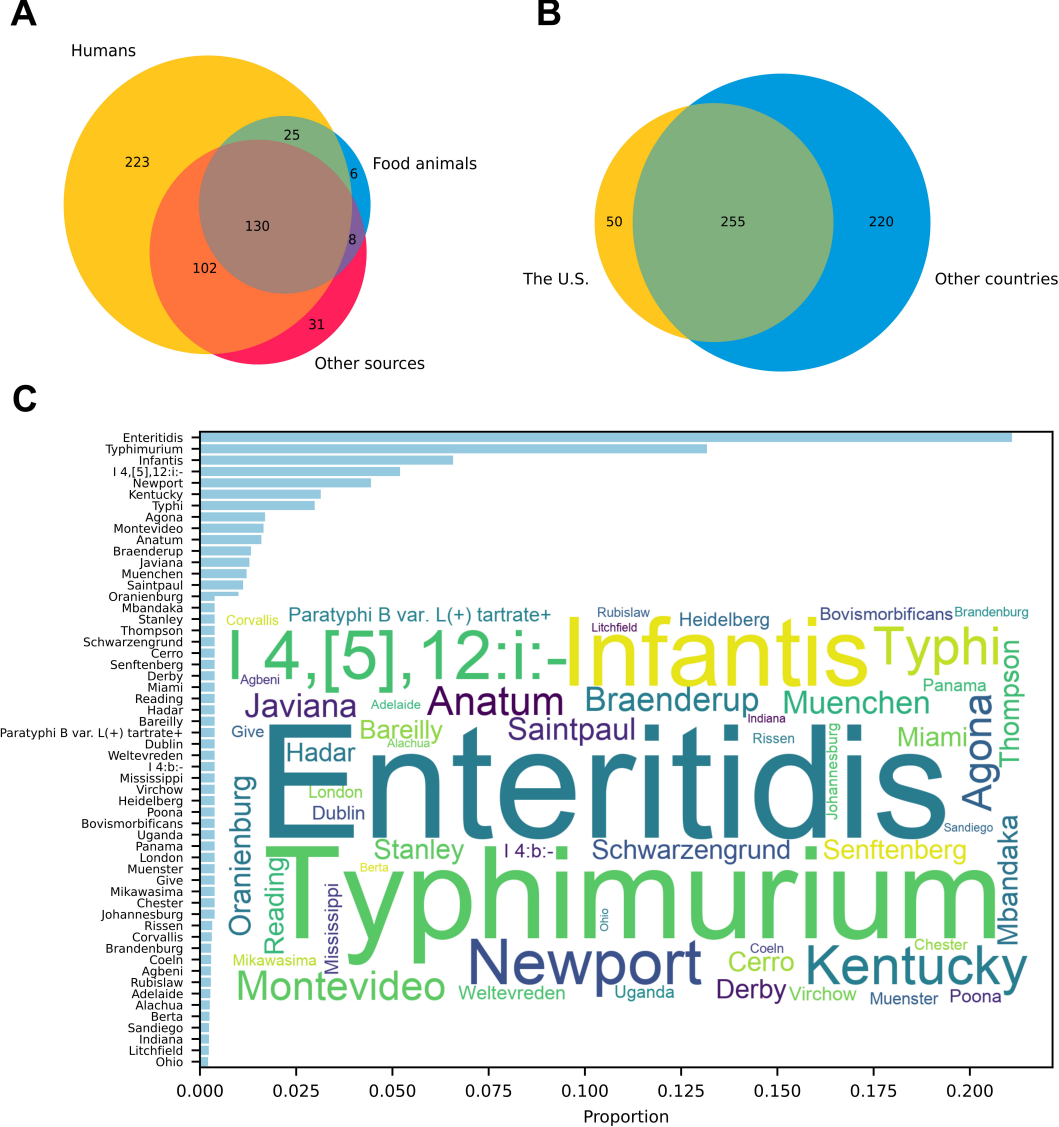
Survey of serotypes from WGS data, 2018 to 2023. (**A**) A Venn diagram showing the distribution of surveyed serotypes among humans, major food animals, and other sources. (**B**) A Venn diagram showing the distribution of surveyed serotypes in the United States and other countries. (**C**) A histogram of the 56 most reported serotypes worldwide that accounted for 90% of surveyed isolates. A word cloud insert highlights these serotypes with font sizes proportional to serotype proportions in the surveyed pool. The data for this figure and percentages of surveyed genomes by serotype are recorded in Table S3.

We surveyed genomes deposited in NCBI Pathogen Detection and EnteroBase between 2018 and 2023. The genomes (*n* = 90,863) represented 525 serotypes from humans, major food animals (poultry, bovine, and swine), and other sources (e.g., environment) were reported worldwide from 2018 to 2023 (Fig. 1A; Table S3). Among these serotypes, each of which included at least two genomes in our data sets, 305 were reported in the United States, 475 were reported from 108 other countries, and 255 were shared between the United States and other countries (Fig. 1B). More serotypes were identified in human isolates (*n* = 480) than in isolates recovered from food animals (*n* = 169) and other sources (*n* = 271), including 223 that were only seen among human isolates (Fig. 1A). Worldwide, the 56 most reported serotypes (Fig. 1C) account for 90% of surveyed isolates (Fig. 1C; Table S3).

### Some serotypes are genetic variants of more common serotypes

**Fig 2:**
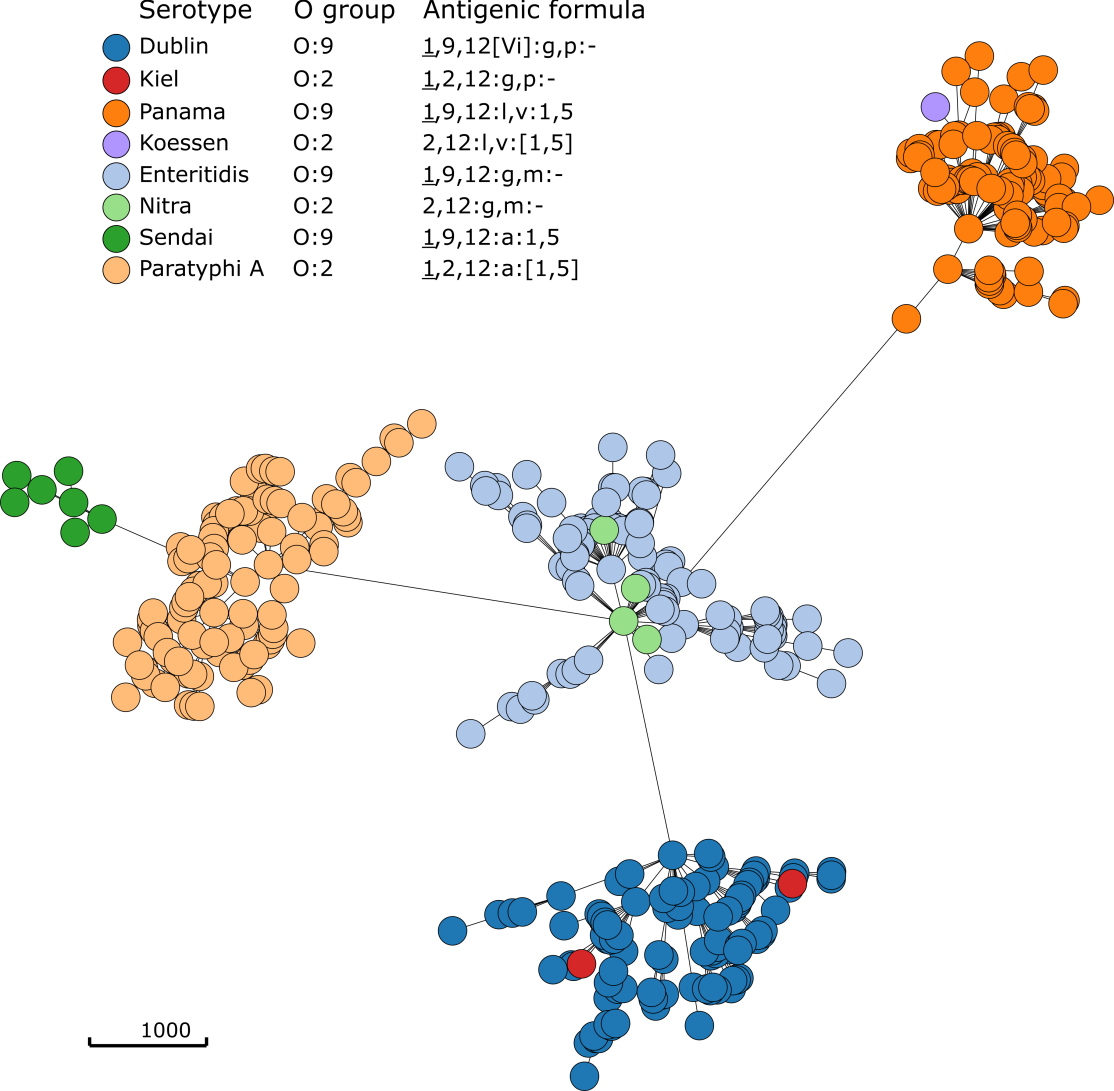
Phylogenetic relationship among representative genomes from serogroups O:2 and O:9. The minimum spanning tree is based on the EnteroBase cgMLST scheme. One hundred isolates of each serotype were randomly selected from EnteroBase. When 100 genomes were not available, all representative isolates were included.

**Fig 3:**
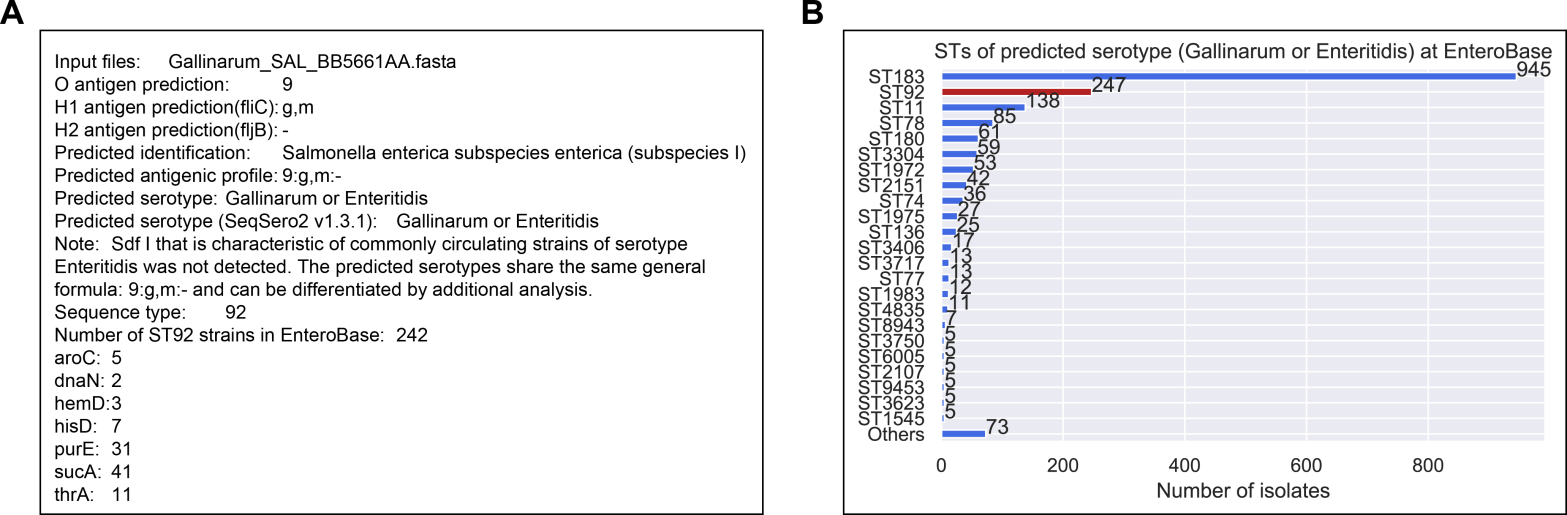
Example of SeqSero2S output for two serotypes that have the same antigenic formula by genetic methods. (A) Textual output of serotype prediction including ST and corresponding 7-gene MLST loci. (B) Distribution of STs among all the “Gallinarum or Enteritidis” predictions by SeqSero2 at EnteroBase. The predicted ST of this query, ST92, is highlighted in red.

Phylogenetic analysis of serogroups O:2 and O:9 serotypes that share the same H antigens revealed a close genetic relationship between serotypes that differ only by O group (Fig. 2). The four variants (Paratyphi A, Nitra, Kiel, and Koessen) belong to serogroup O:2 and are closely related to serogroup O:9 serotypes (Sendai, Enteritidis, Dublin, and Panama, respectively). Closely related serotypes and variants may be differentiated using additional subtyping methods. Seven-gene MLST functionality was added to SeqSero2S to improve subtype information available through the SeqSero2S analysis and to facilitate the identification of possible polyphyletic groups within a serotype, some of which may be a result of the simplified interpretation of serotypes. Figure 3A shows an example of the application of 7-gene MLST data to improve serotype discrimination for serotype Gallinarum. Gallinarum and Enteritidis have the same antigenic formula using genetic methods, I 9:g,m:-. Gallinarum is nonmotile phenotypically and does not express the H: g,m allele. They can be differentiated by phenotypic or genetic tests. SeqSero2S uses *sdf* to identify many Enteritidis strains (23); strains that are I 9:g,m:- and *sdf* negative are indicated as “Gallinarum or Enteritidis.” In this example, the Gallinarum genome was output as serotype “Gallinarum or Enteritidis” because of the absence of *sdf*. Its sequence type was determined to be ST92, which is associated with historical Gallinarum strains and distinct from the major ST183 lineage among isolates predicted as “Gallinarum or Enteritidis” by SeqSero2 in EnteroBase (Fig. 3B). Of note, Gallinarum biovar Gallinarum isolates are ST78 whereas Gallinarum biovar Pullorum isolates are ST92.

### Summary of simplification

A total of 178 serotypes were provisionally removed (Table S1). Among these, 84 were excluded because their exact antigenic formula could not be determined, 66 because no alleles or probes were available, 10 because probes were unavailable for O:9,46,27, 14 because O:54 is encoded on a plasmid, and 4 because they could not be differentiated from other serotypes. A total of 58 serotypes were merged into other serotypes (Table S1). The antigenic formulae of 890 serotypes were simplified, but the simplifications did not result in their merging with other serotypes. Removed serotypes, merged serotypes, and simplified antigenic formulas are categorized in Table S1.

## DISCUSSION

SeqSero2 determines serotype from WGS data by targeting genetic determinants responsible for serotype, including subspecies markers and genes involved in the biosynthesis of O and H antigens. This approach maintains continuity with the phenotypic serotypes and achieves robust serotype prediction, including for serotypes that have not previously been characterized by genetic methods, and without consulting surrogate markers that are unrelated to serotype. The effectiveness of this approach has been validated by U.S. federal agencies (CDC, FDA, and USDA FSIS) for routine *Salmonella* surveillance, especially when serotype is the primary or only *Salmonella* subtype, e.g, surveillance systems NARMS and Laboratory-based Enteric Disease Surveillance. Serotype prediction by SeqSero2 is then followed by other WGS-based subtyping methods, such as genome-wide MLST and SNP analyses when desired.

We developed SeqSero2S to address gaps in our understanding of the genetic basis for some serotype antigens, thus limiting the representation of some serotypes and serotype antigens in a DNA-based scheme. The key aspects of the simplification include (i) disregard secondary O antigens that do not affect serotype assignment; (ii) disregard serotypes that are known to be minor genetic variants of more common serotypes and combine with the parent serotype, e.g., serogroup O:2 except serotype Paratyphi A; (iii) disregard secondary O antigens whose genetic basis is unknown; (iv) disregard antigens and antigenic types that are indicated as variables in the WKL Scheme to be consistent with predominant type for that particular serotype, and (v) disregard antigenic types for which an allele is unavailable or actual antigenic type is unknown due to variable epitopes in the antigenic formula. File S1 and Table S2 provide a detailed explanation of how SeqSero2S designates serotypes.

Our simplified interpretation of the WKL Scheme facilitates the adoption of universal typeability by a genetic approach to serotype determination by addressing multiple challenges in replicating a phenotypic scheme.

### Secondary O antigens in groups O:8 and O:6,14

In serogroup O:8, 39 pairs of serotypes differ by antigen O:6 according to the WKL Scheme. Mikoleit et al. characterized strains from five serotype pairs and found that all strains demonstrated variable expression of O:6, suggesting that O:8 serotypes differing by O:6 are not distinct serotypes (24). Also in serogroup O:8, secondary antigen O:20 is present only if the culture is lysogenized by an O:20 converting phage (phage conversion) (10). The genetic basis for O:20 is unknown. In serogroup O:6,14, two serotype-differentiating secondary O antigens O:24 and O:25 may be variably present without relation to lysogenic conversion (10). Their genetic basis is unknown. In the simplified interpretation, serotypes that differed by O:6 and/or O:20 were merged, as were serotyped that differed by O:24 and O:25. When two serotypes are merged, the name of the serotype that was reported first is retained as detailed in Section V, File S1.

### Antigenic types that are phylogenetically nested within more common types (e.g., serogroup O:2, H antigens of the G complex)

Serogroup O:2 serotypes appear to be closely related to O:9 serotypes with the same H antigens (Fig. 2). The genetic basis for serogroup O:2 in serotype Paratyphi A, a frameshift mutation in an otherwise serogroup O:9 *rfb* region, has been described (25). The genetic basis for the other serotypes has not been described but may be due to similar mutations that result from immune pressure or genetic drift. Similarly, several antigenic types within the G complex are known to be minor genetic variants of more common types, e.g., H:g,m,q, H:g,q, H:g,z63, and H:g,z85 are minor variants of H:g,m in what is otherwise a serotype Enteritidis strain (26). Disregarding these antigenic types helps improve the clarity of *Salmonella* surveillance data by avoiding the confusion caused by closely related serotypes such as Enteritidis and Nitra.

### Antigens and epitopes that are indicated as variables in the WKL Scheme

Some antigens in the WKL Scheme are indicated in square brackets or parentheses, indicating that they are variably present or may be weakly expressed, respectively. Such variability is difficult to represent in a genetic scheme. Furthermore, currently circulating strains typically do not show this variability, making the inclusion of variable epitopes in antigenic formulas confusing in genetic serotype prediction. For the simplification, we disregarded this variability when a definitive antigenic type was known for a particular serotype, e.g., the antigenic formula for serotype Montevideo is I 6,7,14,[54]:g,m,[p],s:[1,2,7] but currently circulating strains are predominantly I 6,7:g,m,s:-.

### Lack of alleles for some antigenic types

Alleles are not available for some antigenic types described in the WKL Scheme, e.g., H:z42, H:g,s,q, H:1,2,5. Also, some antigenic types could not be verified using well-characterized strains. In those instances, the serotype is indicated as “provisionally removed from the scheme” in Table S2 to indicate that SeqSero2S cannot identify that serotype.

### Monophasic variants

Like SeqSero2, SeqSero2S correctly identifies monophasic variants when the *fljB* gene is deleted. For instance, a common monophasic variant I 4,[5],12:i:-, many of which are variants of Typhimurium, is not recognized in the WKL Scheme but is reported as I 4:i- (after the O group simplification) with a note indicating it is a potential variant of Typhimurium. Similarly, I 4:b:- is reported and noted as a potential monophasic variant of Paratyphi B. When a monophasic variant results from a lack of expression or a point mutation, the gene is still present and both SeqSero2 and SeqSero2S predict these strains as the parent serotype. The added MLST functionality of SeqSero2S aids users in interpreting monophasic variants.

To circumvent the incomplete understanding of genetic determinants for some serotypes and to acknowledge that some serotypes are polyphyletic (27), some authors have proposed replacing (26), deducing (27), or affirming (16) serotypes using genetic markers unrelated to serotype. A common surrogate is sequence type (ST) determined by 7-gene MLST (26, 27). Using the correlation between some STs and serotypes, MLST alone was evaluated for serotype prediction for *Salmonella* isolates from routine public health surveillance in the United Kingdom (27). While this method avoided the perceived complexity associated with genetic determinants of serotype and was accurate for commonly circulating strains, misassignment of serotypes occurred when two different serotypes belonged to the same ST (27). In fact, 7-gene MLST was used to show many serotypes were distributed across multiple unrelated phylogenetic clusters (26). Polyphyletic serotypes confound the recognition of evolutionary relatedness by serotype and serve as a main argument to replace serotype with MLST (26). In addition to the polyphyly, another confounding factor of surrogating serotype determination with ST is that some closely related serotypes can share the same ST, such as Paratyphi A and Sendai (ST85). While SeqSero2S incorporates the 7-gene MLST scheme as a complementary analysis, its serotype prediction operates independently without consulting ST. This contrasts with SISTR, which uses core genome MLST to supplement serotype prediction (16).

Genetic determination of serotypes implemented in SeqSero and SeqSero2 is based on the genes responsible for serotypes to replicate traditional serotypes as closely as possible (15, 17). Serotyping is by definition a phenotypic subtyping method; it comes as no surprise that it does not reflect genetic relationships. Modification of the current phenotype-based scheme to try to reflect phylogeny may add to the confusion that already exists regarding *Salmonella* serotypes. Serotyping and MLST, for example, are distinct and complementary subtyping methods. Both can be automatically determined from WGS data, and observations and decisions can be made based on the availability of two complementary subtypes.

We understand the appeal of defining serotypes using additional markers that reflect genetic relatedness; this simplified scheme furthers the goal of concordance of serotypes with natural population structure without the need for additional markers. Furthermore, the WKL Scheme is internationally recognized and accepted. The logistics of developing, obtaining international acceptance, and maintaining a new scheme that includes surrogate genetic markers seems unfeasible. Furthermore, maintaining a concordance between phenotypic and genetic serotypes provides continuity with laboratories that do not have WGS capacity and perform phenotypic serotyping.

The proposed simplification addresses multiple needs in the era of genomic prediction of serotypes. First, it addresses the fact that some serotypes in the WKL Scheme cannot be fully or accurately described using genetic methods. Second, the proposed simplification can be implemented under the current version of the WKL Scheme without publishing a new version of the scheme. Historical data can easily be updated to the simplified scheme if desired. Third, the proposed simplified scheme remains compatible with phenotypic serotyping, allowing continued determination of serotypes by phenotypic methods where needed. Finally, like the phenotypic scheme, this genetic scheme can be updated as recognized serotypes are further characterized or new antigenic types are discovered. This genetic scheme, along with the WKL Scheme, will continue to be improved as new studies enhance our understanding of antigenic diversity and the biological basis for serotype antigens.

A potential drawback of this approach is that merged serotypes may be common in some parts of the world or they may become important in the future, warranting their specific recognition. In the survey of worldwide *Salmonella* genomic surveillance between 2018 and 2023 (Fig. 1), 90% of genomes belonged to 56 serotypes, none of which are affected by the simplification. The simplified scheme can be updated as needed if new data warrants new or modified serotype definitions. Another potential drawback is the removal of secondary O antigens which may result in the merger of distinct genetic lineages into a single “serotype.” The current scheme already describes many serotypes that contain multiple genetic lineages (26). For example, serotype Kentucky contains two distinct lineages; ST152 is commonly found in poultry in the United States and an MDR lineage of ST198 has been associated with human infections in other parts of the world (28, 29). The simplified scheme may add a few more serotypes to that list. Also, when minor variants of a particular antigenic type are merged with the more common type, possible outbreaks might be missed in surveillance data unless that strain has other distinguishing characteristics such as 7-gene MLST or cgMLST type. We added 7-gene MLST analysis to SeqSero2S, including presenting prevalent STs of the predicted serotype, to facilitate the identification of polyphyletic serotypes. Finally, the removal of secondary antigens from the scheme does not preclude continued characterization of them, if desired. The difference is they are no longer required to assign a “serotype” in SeqSero2S. For example, O:5 is an ancillary antigen found in some group O:4 serotypes; *oafA* has been reported to be responsible for this epitope. O:5 has been used to differentiate within serotype Typhimurium, O:5- variants were described as “var. Copenhagen.” Some, but not all, O:5- variants have an *oafA* allele that contains a 7 bp deletion that inactivates the gene (30). These variants are identified as we previously implemented (17).

## MATERIALS AND METHODS

### Survey of circulating serotypes using genomic surveillance data

We surveyed “computed types” (SeqSero2 predicted serotypes) of 32,499 genomes from *Salmonella* isolates deposited at NCBI Pathogen Detection (https://www.ncbi.nlm.nih.gov/pathogens/isolates/#taxgroup_name:%22Salmonella%20enterica%22) from 2018 to 2023, excluding 972 genomes with no or incomplete antigen predictions. These genomes were from major U.S. contributors including PulseNet USA (https://www.cdc.gov/pulsenet/index.html, human isolates from sporadic and outbreak cases), the National Antimicrobial Resistance Monitoring System for Enteric Bacteria (https://www.cdc.gov/narms/index.html; isolates from humans, retail meats, and food animals), and GenomeTrakr (https://www.fda.gov/food/whole-genome-sequencing-wgs-program/genometrakr-network, isolates from non-human domestic and imported sources [food, food facilities, animals, farms, water, etc.]). Only genomes whose location of isolation was the United States were included, although some of these isolates might have been sampled from imported commodities or patients who contracted *Salmonella* abroad. Similarly, we surveyed SeqSero2-predicted serotypes of 58,364 isolates deposited at EnteroBase (https://enterobase.warwick.ac.uk/species/index/senterica) from 2018 to 2023 that did not overlap with the 32,499 genomes from NCBI and came from 108 countries. Similarly, 29,526 EnteroBase genomes with no or incomplete antigen predictions by the EnteroBase implementation of SeqSero2 (genome assembly mode, version <v1.2.1) were excluded. Finally, we excluded 201 serotypes that were represented by only one isolate in NCBI and EnteroBase combined.

### Simplified interpretation of the WKL Scheme

A detailed description of the simplified interpretation of the serotype is presented in File S1. When the simplified interpretation resulted in merging two or more serotypes, we retained the name of the earliest described serotype (Table S1). Simplification rationales are detailed case-by-case in the Discussion.

### SeqSero2S

We implemented the simplification plan in a bioinformatic pipeline termed SeqSero2S. Antigenic types recognized by SeqSero2S are listed in Table S2. We added the 7-gene MLST (31) to SeqSero2S as a separate, complementary subtyping method. The draft or complete assembly of a query genome was scanned against the PubMLST scheme (32) using https://github.com/tseemann/mlst to determine its ST in addition to its serotype. ST data were analyzed in comparison to SeqSero2-predicted serotypes for over 400,000 *Salmonella* isolates deposited in EnteroBase (33) as of May 2023.

## SUPPLEMENTAL MATERIAL

File S1 aem.02600-24-s0001.docx Conventions for simplified serotype designations using genetic methods implemented in SeqSero2S (SS2S).

Table S1 aem.02600-24-s0002.xlsx Simplified scheme.

Table S2 aem.02600-24-s0003.xlsx Antigenic types detected by SeqSero2.

Table S3 aem.02600-24-s0004.xlsx Serotypes surveyed from NCBI Pathogen Detection and EnteroBase.

ASM does not own the copyrights to Supplemental Material that may be linked to, or accessed through, an article. The authors have granted ASM a non-exclusive, world-wide license to publish the Supplemental Material files. Please contact the corresponding author directly for reuse.
