# Supplementary material for: Salmonella serotypes in the genomic era: simplified Salmonella serotype interpretation from DNA sequence data
Source: Appl Environ Microbiol. 2025 Feb 24;91(3):e02600-24. doi: 10.1128/aem.02600-24 (PMC11921320; doi:10.1128/aem.02600-24)
Supplement: File S1 — Conventions for simplified serotype designations using genetic methods implemented in SeqSero2S (SS2S). [file aem.02600-24-s0001.docx]

**Supplemental File 1.**

**Conventions for simplified serotype designations using genetic methods implemented in SeqSero2S (SS2S).**

The simplification is based on the scheme and serotypes presented in the ninth edition of the White-Kauffmann-Le Minor scheme (WKL) (1) including two updates (2, 3). The tables presented are examples and not a comprehensive list. See Supplementary Table 2 for a comprehensive list of serotypes and simplifications.

Serotype in *Salmonella* is based on two surface structures, O antigen and H antigen. O antigen is a carbohydrate antigen and the outermost portion of the cell wall lipopolysaccharides. H antigen is a protein antigen and the filament portion of the bacterial flagella (i.e., flagellin). We categorize O antigens as O group antigens and secondary O antigens. The O group antigen is the core portion of the O antigen, many of the genes responsible for its biosynthesis are located in the *rfb* region. Secondary O antigens are typically side sugars added to the core structure; they are commonly encoded on mobile genetic elements. Many but not all *Salmonella* have two flagellins that are expressed by a phase variation mechanism. The two different flagellar antigenic types are termed Phase 1 and Phase 2. One gene is located in a flagella biosynthesis operon common to all *Enterobacteriaceae*, the other is located in a variable portion of the *Salmonella* genome.

**I. O antigens**

**1) Secondary O antigens that do not affect serotype designation.**

Some secondary O antigens are listed in all serotypes of a particular O group. Others may vary within a serotype but do not differentiate between serotypes. Those antigens are disregarded, resulting in a simplified antigenic formula (Table SF1).

| **Table SF1. Secondary O antigens that do not affect serotype designation** | |
| --- | --- |
| **Secondary O antigen** | **O groups affected** |
| 1 | 2; 4; 9; 13; 6,14; 40; 42; 44; 47; 51; 53; 1,3,19; 9,12,46 |
| 5* | 4 |
| 6 | 7; 8 |
| 12 | 2; 4; 9 |
| 14 | 7; 18 |
| 20 | 8 |
| 27^#^ | 4 |
| **Secondary O antigen** | **O groups affected** |
| 10; 15; 15,34 | 3,10; 1,3,19 |
| 24; 25 | 6,14 |

*Secondary O antigen 5 can be a useful epidemiologic marker for a few serotypes, particularly serotype Typhimurium, antigenic formula I 1 ,4,[5],12:i:1,2. SeqSero2 (SS2) and SS2S have one marker that detects certain O:5- strains (see discussion in the main text). When detected, this information is included as a note in the SS2 and SS2S output, but it is not represented in the antigenic formula.
^#^ O antigen 27 is not technically a secondary O antigen. Itwas originally described to be phage-encoded and subsequently shown to be encoded by a distinct *rfb* region (23), indicating that O27+ and O 27- strains of group O:4 serotypes possess different *rfb* regions. This in not currently reflected in the WKL Scheme.

**2) Secondary O antigens that affect serotype designation.**

**O:6 and O:20 in serogroup O:8**. Secondary O antigen 6 in serogroup O:8 has been shown to be variably expressed (4). This makes it impossible to differentiate between O:6,8 and O:8 serotypes. O:6 in serogroup O:8 is disregarded, and serotypes differentiated by O:6 are merged. Secondary antigen O:20 in serogroup O:8 is thought to be phage-encoded (1), but a genetic marker for this antigen has not been described. O:20 in serogroup O:8 is disregarded and serotypes differentiated by O:20 are merged. Table SF2 presents examples.

| **Table SF2. Examples of merged serotypes in serogroup O:8** | | | | | | | |
| --- | --- | --- | --- | --- | --- | --- | --- |
| **Formula after simplification** | **Serotype after simplification** | **Serotype,**  **WKL Scheme** | **Sub-species** | **O Group, WKL Scheme** | **O Antigen, WKL Scheme** | **H antigen Phase 1, WKL Scheme** | **H antigen Phase 2, WKL Scheme** |
| I 8:d:1,2 | Muenchen | Muenchen | I | 8 | 6,8 | d | 1,2 |
| I 8:d:1,2 | Muenchen | Virginia | I | 8 | 8 | d | 1,2 |
| I 8:e,h:1,2 | Newport | Bardo | I | 8 | 8 | e,h | 1,2 |
| I 8:e,h:1,2 | Newport | Newport | I | 8 | 6,8,20 | e,h | 1,2 |
| I 8:z10:e,n,x | Hadar | Hadar | I | 8 | 6,8 | z10 | e,n,x |
| I 8:z10:e,n,x | Hadar | Istanbul | I | 8 | 8 | z10 | e,n,x |
| I 8:b:1,5 | Nagoya | Korbol | I | 8 | 8,[20] | b | 1,5 |
| I 8:b:z6 | Banalia | Tounouma | I | 8 | 8,[20] | b | z6 |

**O:24 and O:25 in serogroup O:6,14.** ~~S~~ome O:6,14 serotypes cannot be definitively identified based on the KWS because of conflicts in the scheme, e.g., serotypes Lindern and Charity, where O:24 and O:25 are both in square brackets indicating that those epitopes may or may not be present (Table SF3). It is impossible to assign a strain to one serotype or another when O:24 or O:25 is not detected. Also, probes for O:24 and O:25 have not been described. O:24 and O:25 in serogroup O:6,14 are disregarded, and serotypes differentiated by O:24 and O:25 are merged. Table SF3 presents examples.

| **Table SF3. Examples of merged serotypes in serogroup O:6,14** | | | | | | | |
| --- | --- | --- | --- | --- | --- | --- | --- |
| **Formula after simplification** | **Serotype after simplification** | **Serotype, WKL Scheme** | **Sub-species** | **O Group, WKL Scheme** | **O Antigen, WKL Scheme** | **H antigen Phase 1, WKL Scheme** | **H antigen Phase 2, WKL Scheme** |
| I 6,14:d:1,7 | Florida | Florida | I | 6,14 | 1,6,14,[25] | d | 1,7 |
| I 6,14:d:1,7 | Florida | Midway | I | 6,14 | 6,14,24 | d | 1,7 |
| I 6,14:d:e,n,x | Charity | Lindern | I | 6,14 | 6,14,[24] | d | e,n,x |
| I 6,14:d:e,n,x | Charity | Charity | I | 6,14 | 1,6,14,[25] | d | e,n,x |
| I 6,14:e,h:1,5 | Onderstepoort | Bahrenfeld | I | 6,14 | 6,14,[24] | e,h | 1,5 |
| I 6,14:e,h:1,5 | Onderstepoort | Onderstepoort | I | 6,14 | 1,6,14,[25] | e,h | 1,5 |
| I 6,14:y:1,7 | Carrau | Madelia | I | 6,14 | 1,6,14,25 | y | 1,7 |
| I 6,14:y:1,7 | Carrau | Carrau | I | 6,14 | 6,14,[24] | y | 1,7 |

**O:22 and O:23 in serogroup O:13**. Markers for O:22 and O:23 are in SS2 (5), and these secondary O antigens will continue to be detected in SS2S. A genetic marker for O:22 vs O:23 was identified in the O:13 *rfb* region (a *galE* allele NZ [LS483489.1](https://www.ncbi.nlm.nih.gov/nuccore/NZ_LS483489.1?report=genbank) for O:22 and a *galE* allele [NZ_CP029041.1](https://www.ncbi.nlm.nih.gov/nuccore/NZ_CP029041) for O:23). Note that the SeqSero2 outputs only the O:13 group and does not indicate O:22 vs O:23 in the antigenic formula. This has been corrected in SS2S; it interprets both the correct antigenic formula and serotype for these Group O13 pairs.

**3) Serotypes in serogroup O:2**

The *rfb* region in serotype Paratyphi A (antigenic formula I 1,2,12:a:[1,5]) has been shown to contain a frameshift mutation in what is otherwise a serogroup O:9 *rfb* region (6). The mutation is in *tyv*, which is required for the conversion of paratose to tyvelose, a side sugar in the O:9 O group antigen. When *tyv* is inactivated, paratose is incorporated instead, resulting in the serogroup O:2 phenotype.

The other three serotypes in serogroup O:2, Nitra, Kiel, and Koessen, have not been characterized in detail yet, but they also appear to have various frameshift or nonsynonymous substitutions (data not shown, manuscript in preparation). They are also known to be closely genetically related to a serogroup O:9 serotype with the same H antigens (see main text). Serotypes Nitra, Kiel, and Koessen are merged with their parent serogroup O:9 serotype (Enteritidis, Dublin, and Panama, respectively). Serotype Paratyphi A, which is clinically important as a typhoidal serotype (7) is retained.

**4) Serogroup O:54**

Serogroup O:54 is a provisional O group in the WKL Scheme because it is encoded on a plasmid (8, 9) that can be lost (9). Further, the plasmid is mobilizable, thus transferable to other strains. We have observed it in serotypes beyond those described in the WKL Scheme (data not shown). Strains with the plasmid still have a chromosomal *rfb* region and are often variants of common serotypes based on chromosomal *rfb*. Serogroup O:54 is disregarded; serotypes are merged with their parent serotype based on the chromosomal O group. Table SF4 presents examples.

| **Table SF4. Examples of disregarded O:54 serogroup** | | | | | | | |
| --- | --- | --- | --- | --- | --- | --- | --- |
| **Formula after simplification** | **Serotype after simplification** | **Serotype, WKL Scheme** | **Sub-species** | **O Group, WKL Scheme** | **O Antigen, WKL Scheme** | **H antigen Phase 1, WKL Scheme** | **H antigen Phase 2, WKL Scheme** |
| I 21:b:e,n,x | Minnesota | Tonev | I | 54 | 21,54 | b | e,n,x |
| I 8:i:z6 | Kentucky | Poeseldorf | I | 54 | 8,[20],54 | i | z6 |
| I 7:k:1,5 | Thompson | Ochsenwerder | I | 54 | 6,7,54 | k | 1,5 |
| I 4:m,t:- | Banana | Newholland | I | 54 | 4,12,54 | m,t | - |

**II. H antigens**

**1) H antigens where an entire Phase 2 H phase is in square brackets.**

As with O antigens, H antigens that are presented in square brackets in the WKL Scheme may or may not be present in a particular strain of that serotype. The entire H antigen may be in square brackets; this is most common for Phase 2 antigens. For common serotypes in this category, most strains do not have a second phase. We hypothesize that strains with a second phase may have been rare lineages that did not become established or were lost over time. Many Phase 2 H antigens that are in square brackets are not found in commonly circulating strains. Those are disregard; examples are in Table SF5. If a strain that possesses the second phase were encountered, it would be identified by its antigenic formula. This approach has the added benefit of flagging a sequence that may be contaminated with a second *Salmonella* genome that might be otherwise thought to be a diphasic strain of a typically monophasic serotype. When the Phase 2 H antigens that are in square brackets are found in commonly circulating strain, they are retained.

| **Table SF5. Examples of Phase 2 antigens in square brackets** | | | | | | | |
| --- | --- | --- | --- | --- | --- | --- | --- |
| **Formula after simplification** | **Serotype after simplification** | **Serotype, WKL Scheme** | **Sub-species** | **O Group, WKL Scheme** | **O Antigen, WKL Scheme** | **H antigen Phase 1, WKL Scheme** | **H antigen Phase 2, WKL Scheme** |
| I 4:f,g:- | Derby | Derby | I | 4 | 1,4,[5],12 | f,g | [1,2] |
| I 4:f,g,s:- | Agona | Agona | I | 4 | 1,4,[5],12 | f,g,s | [1,2] |
| I 4:g,s,t:- | Kingston | Kingston | I | 4 | 1,4,[5],12,[27] | g,s,t | [1,2] |
| I 7:g,m,s:- | Montevideo | Montevideo | I | 7 | 6,7, 14 | g,m,[p],s | [1,2,7] |
| I 7:g,s,t:- | Menston | Menston | I | 7 | 6,7 | g,s,[t] | [1,6] |

**2) H antigens with individual epitopes in square brackets or in parentheses.**

In some serotypes, individual epitopes of an H antigen are in square brackets or in parentheses. As with O antigens, square brackets mean the epitope may or may not be present; parentheses mean the epitope may be weakly expressed. This variability may be due to multiple alleles that produce different antigenic combinations; this type of variability should be detectable in a genetic method if the alleles are sufficiently different, and probes exist to detect them. Alternatively, the variability may be due to the variability and specificity of the antisera reagents used to phenotypically serotype the strains, or to other biological factors that affect antigen expression. This type of variability is difficult to detect in a genetic method. In most/all instances where we have both phenotypic and genetic serotype data, common serotypes seem to have one antigenic type and related alleles. For the simplification, we described the serotype using the H antigenic type determined by phenotypic serotyping when known or removing any brackets or parentheses if exact antigenic type for commonly circulating types in unknown.

**3) Antigenic types that are minor genetic variants of more common H antigens.**

Substantial diversity has been described for G Complex H antigens. Some types are variants of more common types, e.g., H antigens g,m,q, g,q, g,z63, and g,z85 are variants of H:g,m. Further, these antigens were found in one serotype each, all of which are variants of serotype Enteritidis (10). Similarly, H antigens g,p,s and g,p,u are variants of H:g,p, there is one serotype of each; both are variants of serotype Dublin (11). Antigenic types that are known to be minor variants of more common H antigens are disregarded in the simplification. Examples are in Table SF6.

| **Table SF6. Examples of antigenic types that are variants of more common antigenic types** | | | | | | | |
| --- | --- | --- | --- | --- | --- | --- | --- |
| **Formula after simplification** | **Serotype after simplification** | **Serotype, WKL Scheme** | **Sub-species** | **O Group, WKL Scheme** | **O Antigen, WKL Scheme** | **H antigen Phase 1, WKL Scheme** | **H antigen Phase 2, WKL Scheme** |
| I 9:g,m:- | Enteritidis | Enteritidis | I | 9 | 1,9,12 | g,m | - |
| I 9:g,m:- | Enteritidis | Blegdam | I | 9 | 9,12 | g,m,q | - |
| I 9:g,p:- | Dublin | Dublin | I | 9 | 1,9,12,[Vi] | g,p | - |
| I 9:g,p:- | Dublin | Naestved | I | 9 | 1,9,12 | g,p,s | - |
| I 9:g,p:- | Dublin | Rostock | I | 9 | 1,9,12 | g,p,u | - |
| I 9:g,m:- | Enteritidis | Moscow | I | 9 | 9,12 | g,q | - |
| I 9:g,m:- | Enteritidis | Antarctica | I | 9 | 9,12 | g,z63 | - |
| I 9:g,m:- | Enteritidis | Rosenberg | I | 9 | 9,12 | g,z85 | - |

**4) Third Phases and R Phases**

Some serotypes are described as having a third H antigen type, listed in the “Other” column in the WKL Scheme. Some third-phase antigens are also found in Phase 1 or Phase 2; others are not. The few third phases that have been characterized include flagellar antigen j (12, 13), z66 (14), and d (12, 15). H:j is a variant of *fliC* H:d described in serotype Typhi and recognized in other isolates; it resulted from a 261-nucleotide deletion in gene *fliC*. H:z66 is encoded on a linear plasmid and has been reported in serotype Typhi (24), and there is no probe for H:z66. H:j and H:z66 are disregarded.

H:d is located on a plasmid (22) and has been found in multiple serotypes beyond those described in the WKL Scheme (15, unpublished data). SS2 and SS2S detect all three antigen types in strains possessing a third phase H:d, but report only two flagellar types, the two with the best homology. This can result in an incorrect serotype call. Where possible, serotype designations were updated to recognize H:d as a possible third-phase antigen. Third phases are disregarded in the simplification; however, users should be aware that H:d located on a plasmid may be detected in serotypes beyond those identified in the WKL Scheme and may confound serotype determination in SeqSero2 and SeqSero2S. The contamination detection tool in SS2 and SS2S can be used to investigate the possibly that a strain has a third flagellar antigen.

R Phases are described as “abnormal specificities” in the WKL Scheme and are also listed in the “Other” column (1). They are typically variably present (i.e., presented in square brackets], and appear in addition to the more common Phase 1 and 2 antigens. Further, the genetic basis for R phases is unknown. R Phases are disregarded in the simplification.

**III. Serotypes that are biotypes or pathotypes.**

A small number of serotypes are defined by phenotypic characteristics in addition to serotype antigens (1). Most of these are disregarded. The exceptions are serotypes Paratyphi B (antigenic formula I 1,4,[5],12:b:1,2) and serotypes with antigenic formula I 7:c:1,5 (discussed in section VII below).

Two pathotypes have been described for serotype Paratyphi B, a gastrointestinal pathotype, sometimes referred to as Paratyphi B var. Java or Paratyphi B var. L(+) tartrate+, and a typhoidal pathotype (16). Phenotypically, the typhoidal pathotype is differentiated from the gastrointestinal pathotype by the inability to ferment tartrate. Genetic methods have been developed to differentiate the two pathotypes (17); this marker was incorporated into SS2 (5). The pathotypes can also be differentiated by 7-gene MLST. To date, the typhoidal pathotype appears to be a single lineage, ST86 and its single locus variants (25); the GI pathotype is diverse and represented by a wide range of STs (26).

**IV. Antigens, alleles, and serotypes that are not detected by SeqSero2 and SeqSero2S**

The genetic basis for some serotype antigens is unknown and/or a marker for that antigen has not been described. As a result, SeqSero2 and SeqSero2S do not have a probe to detect those antigenic types and cannot identify those antigens and serotypes (Table SF7). Antigen types for which there is not a genetic probe in the SeqSero2S antigen databases are disregarded in the simplification.

| **Table SF7. H antigens for which there is no allele in SS2 and SS2S** | | | | |
| --- | --- | --- | --- | --- |
| **H antigenic type** | **# serotypes with this antigenic type in KWS** | **Location of antigenic type in WKL Scheme** | | |
|  |  | **Phase 1** | **Phase 2** | **Other Phases** |
| 1,2,5 | 4 |  | 4 |  |
| 1,6,7 | 2 |  | 2 |  |
| g,s,q | 1 | 1 |  |  |
| g,z63 | 1 | 1 |  |  |
| g,z85 | 1 | 1 |  |  |
| z42 | 78 | 11 | 39 | 28 |
| z54 | 2 |  | 1 | 1 |
| **H antigenic type** | **# serotypes with this antigenic type in KWS** | **Location of antigenic type in WKL Scheme** | | |
|  |  | **Phase 1** | **Phase 2** | **Other Phases** |
| z55 | 2 |  | 1 | 1 |
| z57 | 15 | 1 | 6 | 8 |
| z60 | 5 | 1 |  | 4 |
| z61 | 4 | 1 | 2 | 1 |
| z64 | 3 |  | 2 | 1 |
| z67 | 9 |  | 4 | 5 |
| z68 | 2 |  | 1 | 1 |
| z71 | 1 | 1 |  |  |
| z87 | 3 | 1 | 2 |  |
| z88 | 1 |  | 1 |  |
| z91 | 1 | 1 |  |  |
| **Total** | **135** | **20** | **65** | **50** |

Also, the allele probes that are in SeqSero2 tend to be for more common antigens and more common serotypes; alleles from rarer serotypes may not be present in the SS2 and SS2S antigen databases used to make antigen calls. This is particularly true for subspecies II and subspecies VI alleles that seem particularly divergent from subspecies I. The antigen calling algorithms in SeqSero2 and SeqSero2S do not use a percent identity cut-off for calling an antigen type. This may result in miscalls if the appropriate allele is not in the SeqSero2 antigen database; the closest allele/antigen type will be called even if it is divergent and may not be the correct antigen type. Antigenic types that are disregarded in SS2S may be miscalled as their closest genetic relative or not called at all. A low percent identity for the antigen type that is called And/or a length inconsistent with the expected length may be a clue that the antigen type was incorrectly called.

If the antigenic combination predicted by SS2S is not listed in Table S1, the antigenic formula and serotype fields are populated with the antigenic formula predicted by SS2S. The output also includes a note: “The serotype is not recognized in the simplified scheme and may need to be investigated further. It may be a new serotype, a disregarded serotype that can’t be accurately identified using alleles in SS2S, a contaminated genome sequence, or SS2S error”. Examples of antigenic formulas that are not recognized in the WKL Scheme are indicated in Table S3.

**V. Naming conventions for merged serotypes**

When two serotypes are merged, the name of the serotype that was reported first is retained. If the serotypes were reported in the same year, the name appearing first in an alphabetical list is retained. In most cases, this approach resulted in the perceived “more common” name being retained, e.g. serotypes Newport and Bardo. In one instance, serotypes Albany and Duesseldorf, Albany was retained because it appears to be much more common based on references in PubMed.

**VI. Antigenic formulas for *Salmonella bongori* serotypes**

In the WKL Scheme, the “Type” data field indicates the serotype name for *S. enterica* subspecies *enterica* serotypes and the *S. enterica* subspecies are abbreviated by Roman numeral (i.e., II, IIIa, IIIb, IV, and VI). *S. bongori* serotypes are indicated by “V” in the “Type” data field; “V” corresponds to “subgenera V” which was its designation before being recognized as a separate species of *Salmonella* (17). In the simplification, *S. bongori* antigenic formulas are recognized by not containing a Roman numeral; *S. enterica* antigenic formulas are recognized by starting with the Roman numeral that corresponds to their subspecies (e.g., I for subspecies *enterica*, II for subspecies *salamae*).

**VII. Serotypes that cannot be definitively identified by SS2 and SS2S**

**1) Serotypes with antigenic formula I 7:c:1,5.** Serotypes with antigenic formula I 7:c:1,5 include multiple biotypes/pathotypes (1), in particular, serotypes Paratyphi C and Choleraesuis, which are important human pathogens. These biotypes/pathotypes can be differentiated by phenotypic tests (1) and 7-gene MLST (27); specific markers to differentiate based on genotype have not been described.

**2) Serotypes Enteritidis and Gallinarum.** Serotype Enteritidis (antigenic formula I 9:g,m:- ) is a globally distributed nontyphoidal serotype; serotype Gallinarum, which includes biotype Pullorum, is an important bird pathogen. Phenotypically, Gallinarum is nonmotile (antigenic formula I 9:-:- ) and differentiated from nonmotile variants of other serogroup O9 serotypes by phenotypic profile. Genetically, Gallinarum has a non-expressed H:g,m:- allele, so it is indistinguishable from Enteritidis using genetic methods; both have the antigenic formula I 9:g,m:- in SS2 and SS2S. Many modern strains of Enteritidis, but not all, possess the marker s*df (18)*, which has been used to positively identify Enteritidis by genetic methods, including SeqSero (19), SS2, and SS2S. The mutation leading to nonmotility in Gallinarum has been identified (20); it will be included in a future version of SS2 and SS2S to positively identify Gallinarum strains.

**3) Serotypes Miami and Sendai**

Serotypes Miami and Sendai have the same antigenic formula, I 9:a:1,5, and can be differentiated by phenotypic tests (1). Further, Sendai appears to be related to serotype Paratyphi A and unrelated to serotype Miami ((21); also see main text). Genetic markers are not currently available to differentiate Miami and Sendai. They can be differentiated phylogenetically or by 7-gene MLST; ST80 appears to be a widely distributed lineage of Miami.

**4) Serotypes Abortusequi and Bispebjerg**

Serotypes Abortusequi (I 4:-:e,n,x) has a non-expressed H:a allele, making it impossible to distinguish from serotype Bispejerg (I 4:a:e,n,x) using genetic methods based only on serotype antigens. They can be differentiated phylogenetically or by 7-gene MLST; ST251 appears to represent an Abortusequi lineage (28).

**VII. Probes that may produce incorrect serotype designations.**

A few probes in SS2 are known to produce incorrect results.

The serogroup O:9,46,27 probe cross reacts with some O:9, O:9,46, and O:4,27 strains. Serogroup O:9,46,27 is found exclusively in subspecies II (1). A search of GenBank using the Pathogen Detection Browser (https://www.ncbi.nlm.nih.gov/pathogens/), revealed that O:9,46,27 was detected in 5 subspecies II genomes; the full antigenic formula corresponded with a serotype recognized in the WKL Scheme for only one of the five genomes. O:9,46,27 was detected in an additional 255 genomes, all of which were identified as subspecies I; most appeared correspond to a common serogroup O:4 and O:9 serotypes. See Table S3 for additional examples. The serogroup O:9,46,27 probe was removed from SS2S and will be removed in a future version of SS2.

**References**

1. Grimont PA, Weill FX. Antigenic formulae of the Salmonella serovars, 9th edition. WHO Collaborating Centre for Reference and Research on Salmonella, Institut Pasteur; 2007.

2. Guibourdenche M, Roggentin P, Mikoleit M, Fields PI, Bockemuhl J, Grimont PA, Weill FX. Supplement 2003-2007 (No. 47) to the White-Kauffmann-Le Minor scheme. Res Microbiol. 2010 Jan-Feb;161(1):26-9.

3. Issenhuth-Jeanjean S, Roggentin P, Mikoleit M, Guibourdenche M, de Pinna E, Nair S, et al. Supplement 2008-2010 (no. 48) to the White-Kauffmann-Le Minor scheme. Res Microbiol. 2014 Sep;165(7):526-30.

4. Mikoleit M, Van Duyne MS, Halpin J, McGlinchey B, Fields PI. Variable expression of O:61 in Salmonella group C2. J Clin Microbiol. 2012 Dec;50(12):4098-9.

5. Zhang S, den Bakker HC, Li S, Chen J, Dinsmore BA, Lane C, et al. SeqSero2: Rapid and Improved Salmonella Serotype Determination Using Whole-Genome Sequencing Data. Appl Environ Microbiol. 2019 Dec 1;85(23).

6. Verma NK, Quigley NB, Reeves PR. O-antigen variation in Salmonella spp.: rfb gene clusters of three strains. J Bacteriol. 1988 Jan;170(1):103-7.

7. Crump JA, Mintz ED. Global trends in typhoid and paratyphoid Fever. Clin Infect Dis. 2010 Jan 15;50(2):241-6.

8. Popoff MY, Le Minor L. Expression of antigenic factor O:54 is associated with the presence of a plasmid in Salmonella. Ann Inst Pasteur Microbiol (1985). 1985 Sep-Oct;136B(2):169-79.

9. Keenleyside WJ, Perry M, Maclean L, Poppe C, Whitfield C. A plasmid-encoded rfbO:54 gene cluster is required for biosynthesis of the O:54 antigen in Salmonella enterica serovar Borreze. Mol Microbiol. 1994 Feb;11(3):437-48.

10. Stanley J, Baquar N. Phylogenetics of Salmonella enteritidis. Int J Food Microbiol. 1994 Jan;21(1-2):79-87.

11. Achtman M, Wain J, Weill FX, Nair S, Zhou Z, Sangal V, et al. Multilocus sequence typing as a replacement for serotyping in Salmonella enterica. PLoS Pathog. 2012;8(6):e1002776.

12. McQuiston JR, Parrenas R, Ortiz-Rivera M, Gheesling L, Brenner F, Fields PI. Sequencing and comparative analysis of flagellin genes fliC, fljB, and flpA from Salmonella. J Clin Microbiol. 2004 May;42(5):1923-32.

13. Old DC, Rankin SC, Crichton PB. Assessment of strain relatedness among Salmonella serotypes Salinatis, Duisburg, and Sandiego by biotyping, ribotyping, IS200 fingerprinting, and pulsed-field gel electrophoresis. J Clin Microbiol. 1999 Jun;37(6):1687-92.

14. Baker S, Hardy J, Sanderson KE, Quail M, Goodhead I, Kingsley RA, et al. A novel linear plasmid mediates flagellar variation in Salmonella Typhi. PLoS Pathog. 2007 May 11;3(5):e59.

15. Smith NH, Selander RK. Molecular genetic basis for complex flagellar antigen expression in a triphasic serovar of Salmonella. Proc Natl Acad Sci U S A. 1991 Feb 1;88(3):956-60.

16. Selander RK, Beltran P, Smith NH, Barker RM, Crichton PB, Old DC, et al. Genetic population structure, clonal phylogeny, and pathogenicity of Salmonella paratyphi B. Infect Immun. 1990 Jun;58(6):1891-901.

17. Malorny B, Bunge C, Helmuth R. Discrimination of d-tartrate-fermenting and -nonfermenting Salmonella enterica subsp. enterica isolates by genotypic and phenotypic methods. J Clin Microbiol. 2003 Sep;41(9):4292-7.

18. Agron PG, Walker RL, Kinde H, Sawyer SJ, Hayes DC, Wollard J, Andersen GL. Identification by subtractive hybridization of sequences specific for Salmonella enterica serovar enteritidis. Appl Environ Microbiol. 2001 Nov;67(11):4984-91.

19. Zhang S, Yin Y, Jones MB, Zhang Z, Deatherage Kaiser BL, Dinsmore BA, et al. Salmonella serotype determination utilizing high-throughput genome sequencing data. J Clin Microbiol. 2015 May;53(5):1685-92.

20. Reeves MW, Evins GM, Heiba AA, Plikaytis BD, Farmer JJ, 3rd. Clonal nature of Salmonella typhi and its genetic relatedness to other salmonellae as shown by multilocus enzyme electrophoresis, and proposal of Salmonella bongori comb. nov. J Clin Microbiol. 1989 Feb;27(2):313-20.

21. Feng Y, Lin E, Zou S, Chen CL, Chiu CH. Complete genome sequence of Salmonella enterica serovar Sendai shows H antigen convergence with S. Miami and recent divergence from S. Paratyphi A. BMC Genomics. 2019 May 22;20(1):398.

22. Smith NH and Selander RK. 1991. Molecular genetic basis for complex flagellar antigen expression in a triphasic serovar of Salmonella. Proc. Natl. Acad. Sci. USA 88:956–960.

23. Wang L, Andrianopoulos K, Liu D, Popoff MY, Reeves PR. Extensive Variation in the O-Antigen Gene Cluster within One Salmonella enterica Serogroup Reveals an Unexpected Complex History. J Bacteriol. 2002 Mar; 184(6): 1669–1677

24. Baker S, Hardy J, Sanderson KE, Quail M, Goodhead I, Kingsley RA, Parkhill J, Stocker B, Dougan G. A novel linear plasmid mediates flagellar variation in Salmonella Typhi. PLoS Pathog. 2007 May 11; 3(5):e59.

25. Weill F-X, Frézal L, Tran-Dien A, et al. Genomic perspective on the bacillus causing paratyphoid B fever, 18 June 2024, PREPRINT (Version 1) available at Research Square [https://doi.org/10.21203/rs.3.rs-4502330/v1]

26. Connor TR, Owen SV, Langridge G, et al. What's in a Name? Species-Wide Whole-Genome Sequencing Resolves Invasive and Noninvasive Lineages of Salmonella enterica Serotype Paratyphi B. mBio. 2016 Aug 23;7(4):e00527-16.

27. Achtman M, Wain J, Weill F-X, et al. Multilocus sequence typing as a replacement for serotyping in Salmonella enterica. PLoS Pathog. 2012;8(6):e1002776.

28. Fabre L, Zhang J, Guigon G, et al. CRISPR typing and subtyping for improved laboratory surveillance of Salmonella infections. PLoS One. 2012;7(5):e36995.
